# Supplementary material for: Regulation of Hippo signaling by Atrophin in the developing Drosophila wing
Source: Development. 2026 Aug 5;153(15):dev205540. doi: 10.1242/dev.205540 (PMC13480958; doi:10.1242/dev.205540)
Supplement: Supplementary file 1 [file develop-153-205540-s1.pdf]

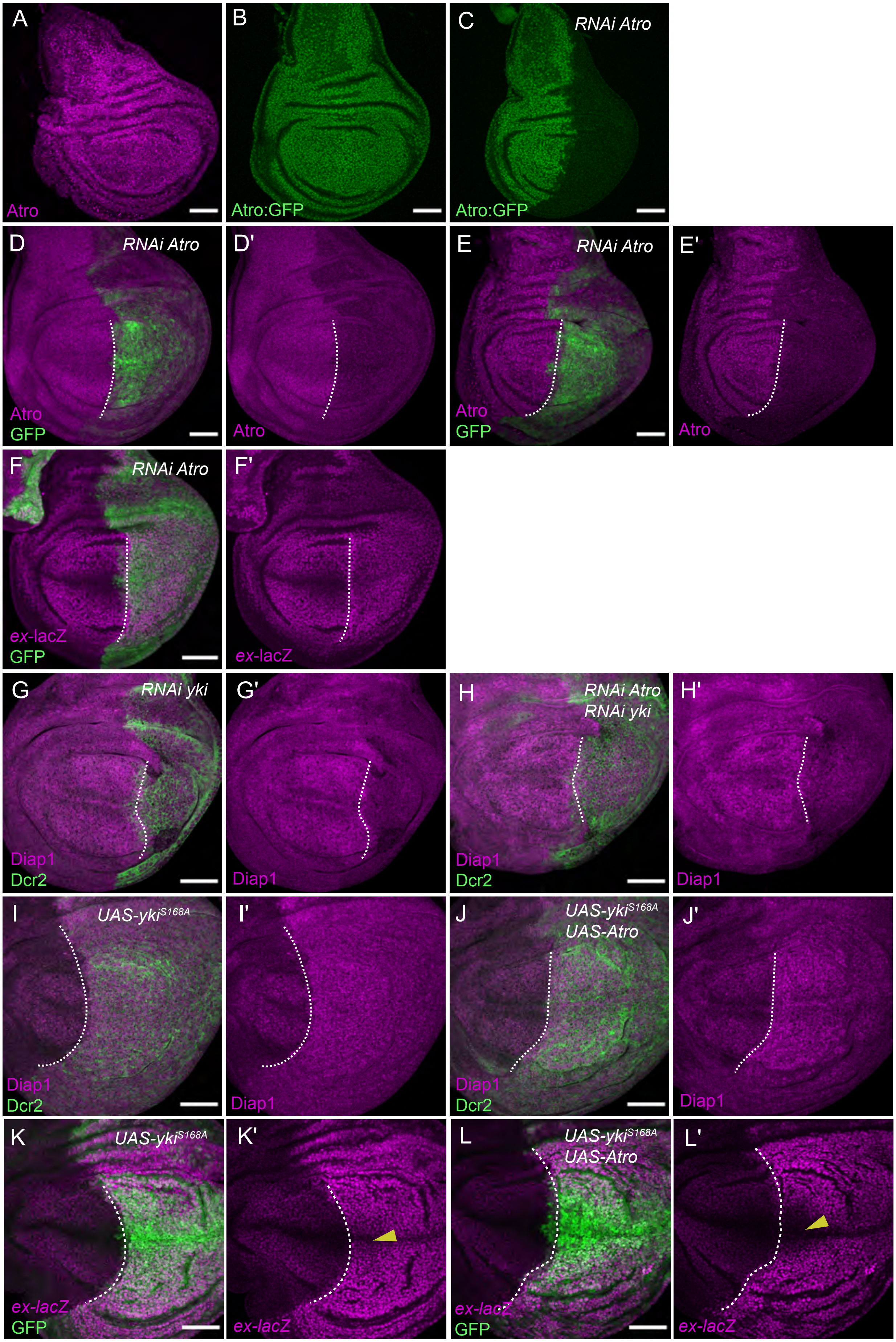
A
Atro
B
Atro:GFP
C
RNAi Atro
Atro:GFP
D
RNAi Atro
Atro
GFP
D'
Atro
E
RNAi Atro
Atro
GFP
E'
Atro
F
RNAi Atro
ex-lacZ
GFP
F'
ex-lacZ
G
RNAi yki
Diap1
Dcr2
G'
Diap1
H
RNAi Atro
RNAi yki
Diap1
Dcr2
H'
Diap1
I
UAS-yki S168A
Diap1
Dcr2
I'
Diap1
J
UAS-yki S168A
UAS-Atro
Diap1
Dcr2
J'
Diap1
K
UAS-yki S168A
ex-lacZ
GFP
K'
ex-lacZ
L
UAS-yki S168A
UAS-Atro
ex-lacZ
GFP
L'
ex-lacZ

Fig. S1. Atro interaction with Yki

A) Expression of Atro protein in wing discs, detected by antibody staining. B) Expression of Atro:GFP from a genomic construct (PBac-Gug-GFP.FPTB). C) Expression of Atro:GFP in wing discs expressing *en-Gal4 tub-Gal80^{ts} UAS-Dcr2* crossed to Atro:GFP *UAS-RNAi-Atro[HMS00756].* D-E) Expression of Atro protein in wing discs expressing *en-Gal4 UAS-GFP ex-lacZ tub-Gal80^{ts} UAS-Dcr2* crossed to *UAS-RNAi-Atro[HMS00756]* (D) and *UAS-RNAi-Atro[KK100675]* (E), stained with mouse (D) or rabbit (E) anti-Atro. F) Expression of *ex-lacZ* (magenta) in third instar wing discs from *en-Gal4 UAS-GFP ex-lacZ; tub-Gal80^{ts} UAS-Dcr2* crossed to *UAS-RNAi-Atro[KK100675]*. Posterior is labeled by GFP (green). G-J) Expression of Diap1 (magenta) in wing discs from *en-Gal4; tub-Gal80^{ts} UAS-Dcr2* crossed to *UAS-RNAi-yki [KK109756]* (G), *UAS-RNAi-yki [KK109756]* and *UAS-RNAi-Atro[HMS00756]* (H*), UAS-yki^{S168A}* (I), *UAS-yki^{S168A}* and *UAS-3xFLAG:Atro* (J). Posterior compartment is labeled by Dcr2 (green). K,L) Expression of *ex-lacZ* (magenta) in wing discs from *en-Gal4 UAS-GFP ex-lacZ; tub-Gal80^{ts} UAS-Dcr2* crossed to *UAS-yki^{S168A}* (K) or *UAS-yki^{S168A}* and *UAS-3xFLAG:Atro* (L). Posterior compartment is labeled by GFP (green). Panels marked by prime symbols show individual channels, dashed lines mark AP boundary, yellow arrowheads point to distal DV boundary region, Scale bars=50 μm.

Development • Supplementary information

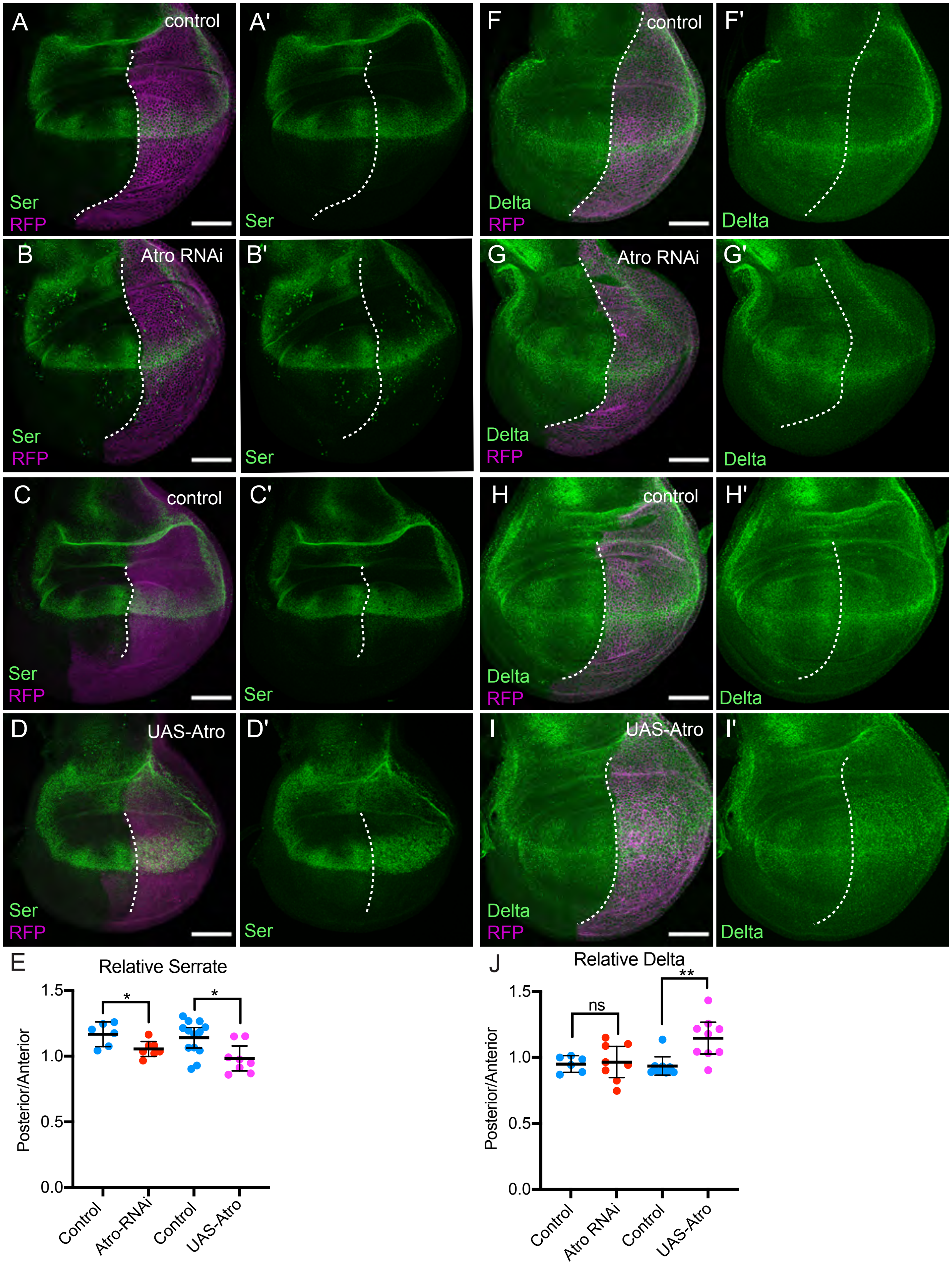

A
control
Ser
RFP
A'
Ser
F
control
Delta
RFP
F'
Delta
B
Atro RNAi
Ser
RFP
B'
Ser
G
Atro RNAi
Delta
RFP
G'
Delta
C
control
Ser
RFP
C'
Ser
H
control
Delta
RFP
H'
Delta
D
UAS-Atro
Ser
RFP
D'
Ser
I
UAS-Atro
Delta
RFP
I'
Delta
E
Relative Serrate
Posterior/Anterior
1.5
1.0
0.5
0.0
*
*
Control
Atro-RNAi
Control
UAS-Atro
J
Relative Delta
Posterior/Anterior
1.5
1.0
0.5
0.0
ns
**
Control
Atro RNAi
Control
UAS-Atro

Fig. S2. Influence of Atro on Notch ligand expression

A-D) Expression of Ser (green) in wing discs from *hh-Gal4 tub-Gal80^{ts}* crossed to OR (control) (A,C), *UAS-RNAi-Atro* (B) and *UAS-3xFLAG:Atro* (D). Posterior compartments are marked by mCD8:RFP stain (magenta), dashed lines mark anterior-posterior compartment boundary, panels marked by prime symbols show individual channels, scale bars=50 μm. (E) Quantitation of Serrate levels in posterior versus anterior cells in control (n=6) versus Atro RNAi (n=7) wing discs, or control (n=13) versus over-expression (n=8) wing discs. F-I) Expression of Delta (green) in wing discs from *hh-Gal4 tub-Gal80^{ts}* crossed to OR (control) (F,H), *UAS-RNAi-Atro* (G) and *UAS-3xFLAG:Atro* (I). (J) Quantitation of Delta levels in posterior versus anterior cells in control (n=6) versus Atro RNAi (n=8) wing discs, or control (n=8) versus Atro over-expression (n=9) wing discs. Significance of differences calculated by unpaired t-tests, error bars indicate mean±c.i.

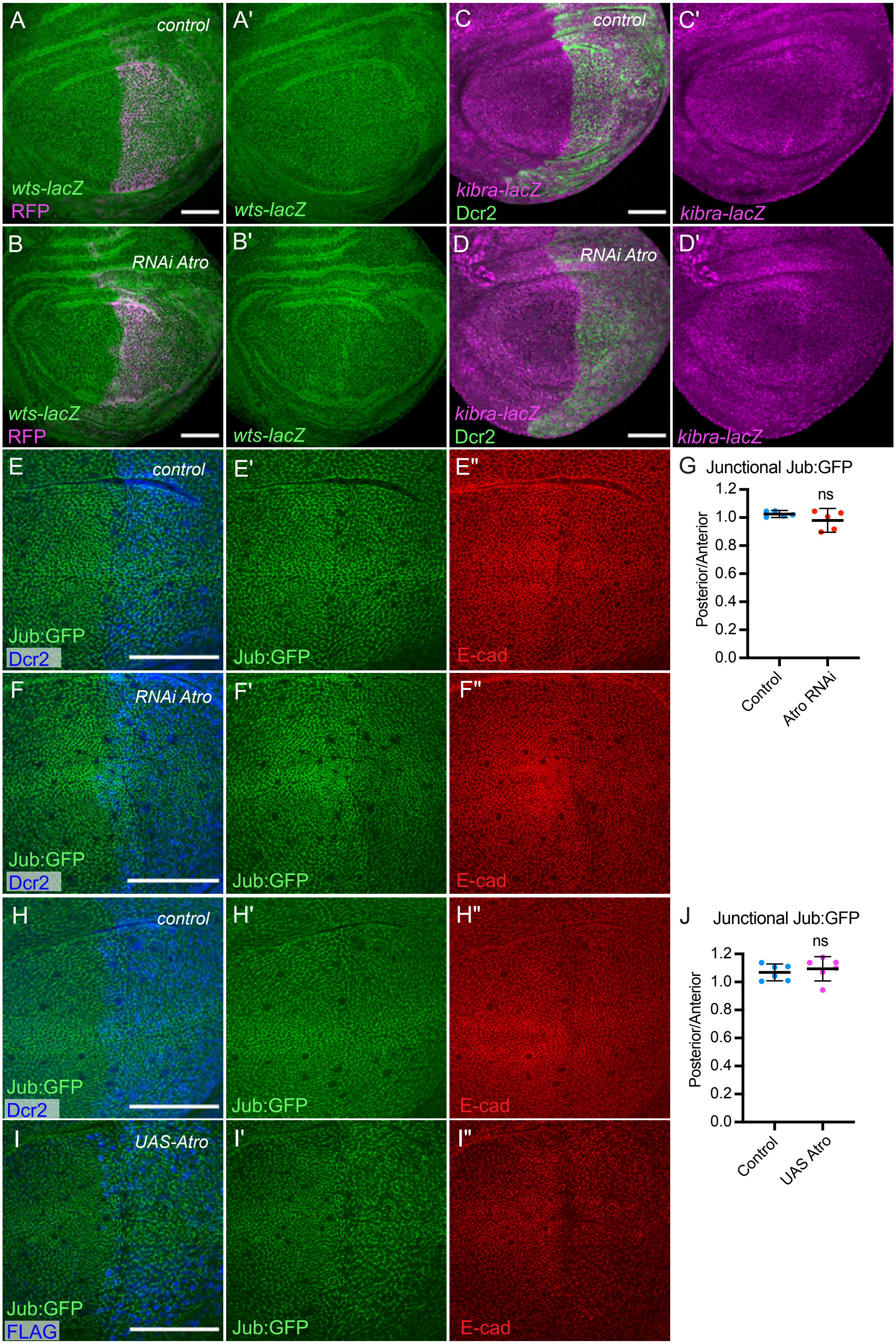
A
control
wts-lacZ
RFP
A'
wts-lacZ
C
control
kibra-lacZ
Dcr2
C'
kibra-lacZ
B
RNAi Atro
wts-lacZ
RFP
B'
wts-lacZ
D
RNAi Atro
kibra-lacZ
Dcr2
D'
kibra-lacZ
E
control
Jub:GFP
Dcr2
E'
Jub:GFP
E''
E-cad
G
Junctional Jub:GFP
Posterior/Anterior
1.2
1.0
0.8
0.6
0.4
0.2
0.0
ns
Control
Atro RNAi
F
RNAi Atro
Jub:GFP
Dcr2
F'
Jub:GFP
F''
E-cad
H
control
Jub:GFP
Dcr2
H'
Jub:GFP
H''
E-cad
J
Junctional Jub:GFP
Posterior/Anterior
1.2
1.0
0.8
0.6
0.4
0.2
0.0
ns
Control
UAS Atro
I
UAS-Atro
Jub:GFP
FLAG
I'
Jub:GFP
I''
E-cad

Fig. S3. Atro regulation of Yki is not explained by regulation of Wts, Kibra, or Jub

(A,B) *wts-lacZ* expression (green) in wing discs from *en-Gal4 UAS-RFP tub-Gal80^{ts} UAS-Dcr2* crossed to control (OR) (A) or *UAS-RNAi-Atro[KK100675]* (B). Posterior compartment is labeled by RFP (magenta). (C,D) *kibra-lacZ* expression (magenta) in wing discs from *hh-Gal4 tub-Gal80^{ts} UAS-Dcr2* crossed to control (OR) (C) or *UAS-RNAi-Atro[KK100675]* (D). Posterior compartment is labeled by Dcr2 (green). (E,F,H,I) Jub:GFP (green) in wing discs expressing *en-Gal4 tub-Gal80^{ts} jub:GFP UAS-Dcr2* crossed to control (OR) (E), *UAS-RNAi-Atro[HMS00756]* (F), control (OR) (H), or *UAS-3xFLAG:Atro* (I). Posterior compartment is labeled by Dcr2 or FLAG:Atro (blue). Panels marked by prime or double prime symbols show individual channels, scale bars=50 μm. (G,J) Quantitation of relative Jub:GFP/E-cad in posterior versus anterior cells from wing discs of the genotypes in E,F (G) or H,I (J). N=5 (control E), N=8 (Atro RNAi). N=6 (control H), N=6 (UAS-Atro). Significance of differences calculated by unpaired t-test, error bars indicate mean±c.i.

Development • Supplementary information

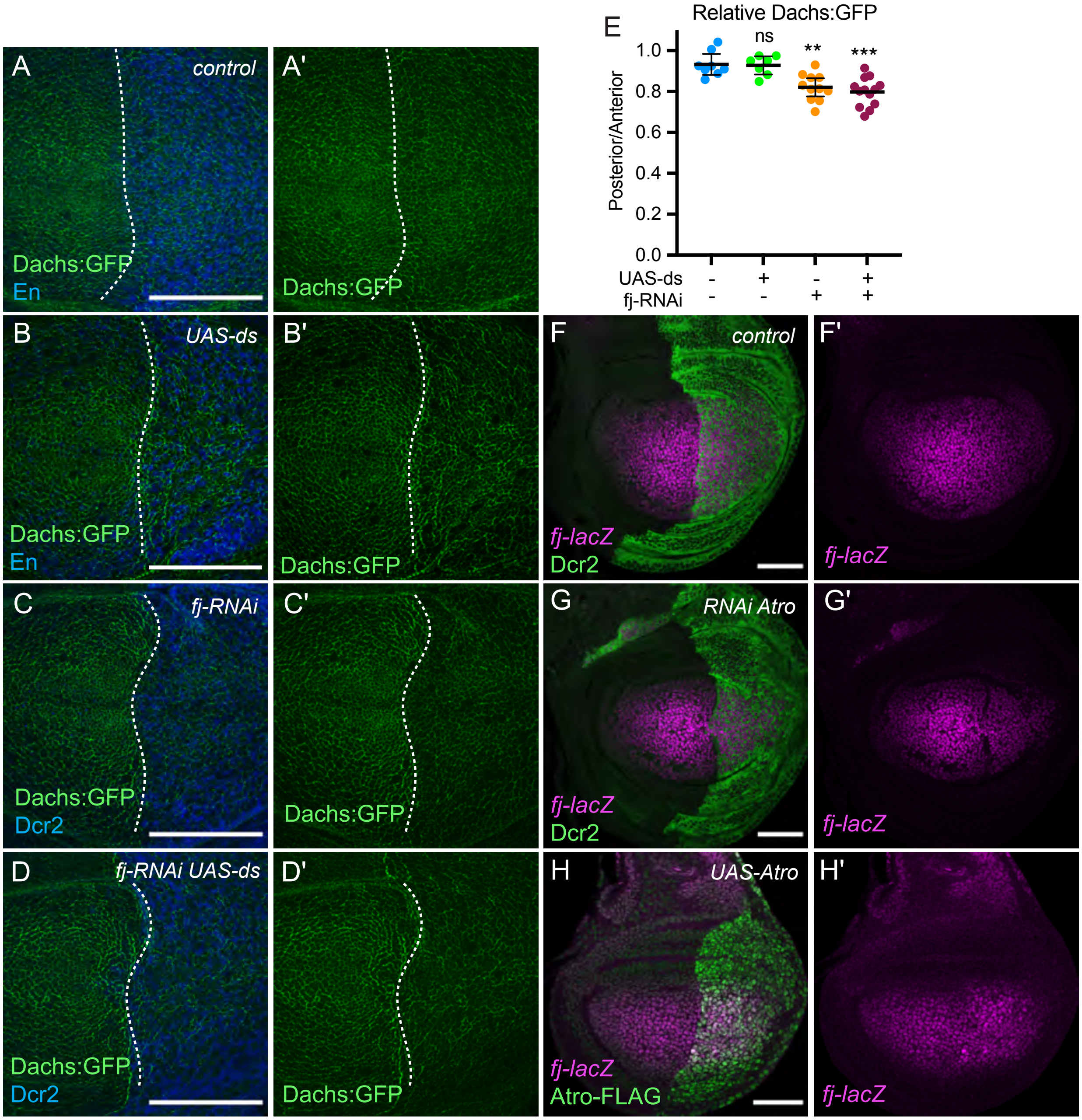
A
control
Dachs:GFP
En
A'
Dachs:GFP
E
Relative Dachs:GFP
Posterior/Anterior
1.0
0.8
0.6
0.4
0.2
0.0
ns
**
***
UAS-ds
fj-RNAi
B
UAS-ds
Dachs:GFP
En
B'
Dachs:GFP
F
control
fj-lacZ
Dcr2
F'
fj-lacZ
C
fj-RNAi
Dachs:GFP
Dcr2
C'
Dachs:GFP
G
RNAi Atro
fj-lacZ
Dcr2
G'
fj-lacZ
D
fj-RNAi UAS-ds
Dachs:GFP
Dcr2
D'
Dachs:GFP
H
UAS-Atro
fj-lacZ
Atro-FLAG
H'
fj-lacZ

Fig. S4. Additional analysis of Fj and Dachs regulation

(A-D) Dachs:GFP in wing discs expressing *dachs:GFP* (control) (A), *en-Gal4 tub-Gal80^{ts} dachs:GFP* crossed to *UAS-ds* (B), *fj-RNAi* (C), or *fj-RNAi UAS-ds* (D). Posterior is labeled by Engrailed (En) or (Dcr2) (blue), panels marked by prime symbols show individual channels, dashed line marks AP boundary, scale bars=50 μm. (E) Quantitation of relative Dachs:GFP in posterior versus anterior cells from wing discs of the genotypes in A-D, control (n=8), fj-RNAi (n=11), fj-RNAi UAS-Atro (n=13), UAS-ds (n=7). Significance of differences calculated by one-way ANOVA, error bars indicate mean±c.i. (F-H) Expression of *fj-lacZ* (magenta) in wing discs expressing *fj-lacZ* hh-*Gal4 tub-Gal80^{ts} UAS-Dcr2* crossed to control (OR) (F), *UAS-RNAi-Atro[HMS00756]* (G), or *UAS-3xFLAG:Atro* (H). Posterior compartment is labeled by Dcr2 or Atro:FLAG (green).

Development • Supplementary information

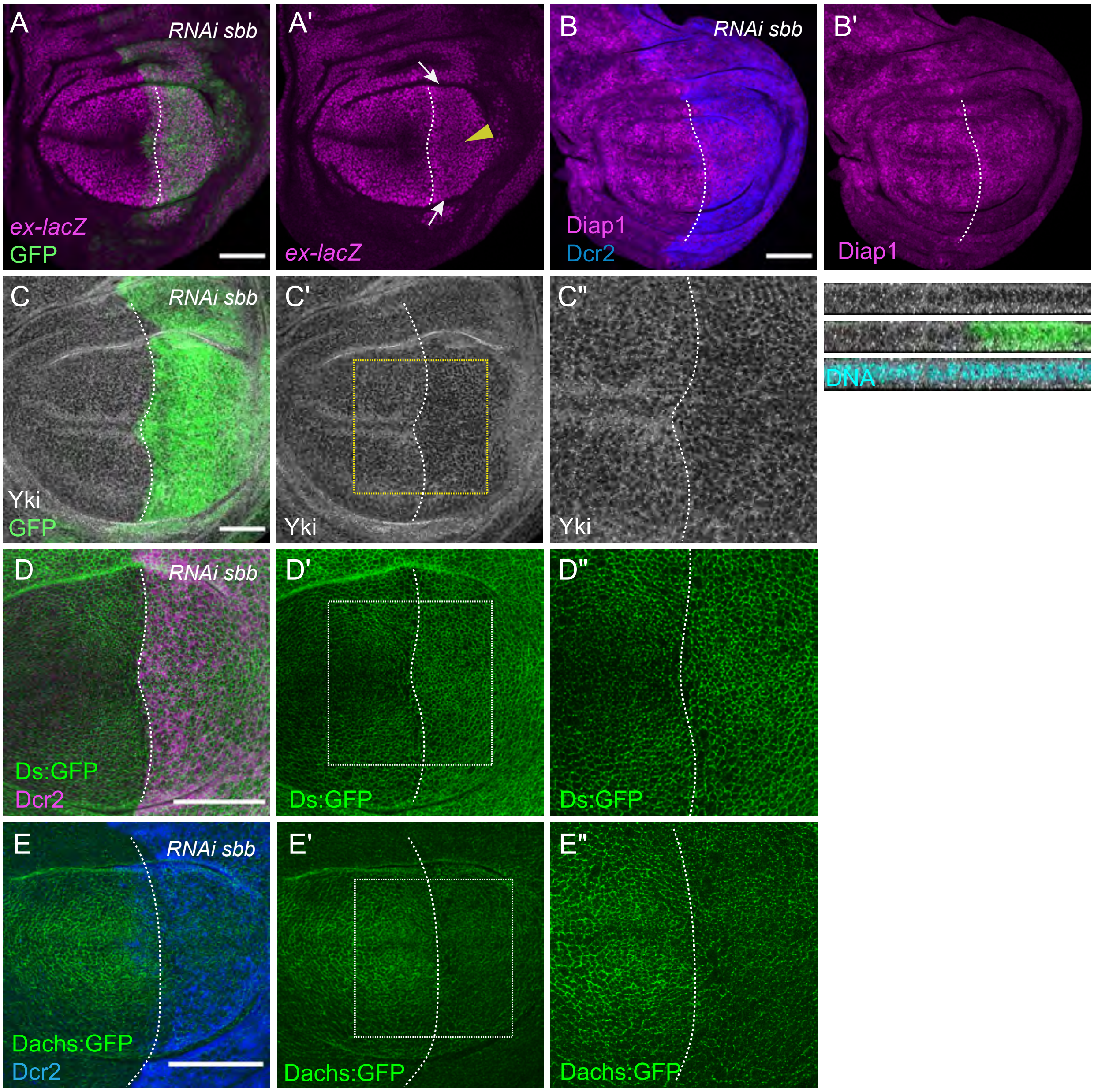
A
RNAi sbb
ex-lacZ
GFP
A'
ex-lacZ
B
RNAi sbb
Diap1
Dcr2
B'
Diap1
C
RNAi sbb
Yki
GFP
C'
Yki
C"
Yki
DNA
D
RNAi sbb
Ds:GFP
Dcr2
D'
Ds:GFP
D"
Ds:GFP
E
RNAi sbb
Dachs:GFP
Dcr2
E'
Dachs:GFP
E"
Dachs:GFP

Fig. S5. Scribbler influences Hippo signaling similarly to Atro

A) Expression of *ex-lacZ* (magenta) in wing discs from *en-Gal4 UAS-GFP ex-lacZ tub-Gal80*^{ts} *UAS-Dcr2* crossed to *UAS-RNAi-sbb*. Posterior compartment is labeled by GFP (green). (B) Expression of Diap1 (magenta) in wing discs from *en-Gal4 tub-Gal80*^{ts} *UAS-Dcr2 dachs:GFP* crossed to *UAS-RNAi-sbb*. Posterior compartment is labeled by Dcr2 (blue). (C) Yki protein (white) in wing discs expressing *en-Gal4 UAS-GFP tub-Gal80*^{ts} *UAS-Dcr2* crossed to *UAS-RNAi-sbb*, with posterior marked by expression of GFP (green). Images at far right of C" show vertical sections through the discs, including Yki, Yki and GFP, Yki and a DNA stain as a nuclear marker (cyan). D) Ds:GFP in wing disc expressing *hh-Gal4 tub-Gal80*^{ts} *UAS-Dcr2 ds:GFP* crossed to *UAS-RNAi-sbb*, with posterior compartment labeled by Dcr2 (magenta). E) Dachs:GFP (green) in wing disc expressing *en-Gal4 tub-Gal80*^{ts} *UAS-Dcr2 dachs:GFP* crossed to *UAS-RNAi-sbb*, with posterior labeled by Dcr2 (blue). Dashed lines mark AP boundary, channels marked by prime symbols show single channel of staining, panels marked by double prime symbols show higher magnification of the boxed regions. Scale bars=50μm.

Table S1. Antibodies used

Reagent	Source	Identifier
Anti-Yki (Rabbit) (1:80)	Irvine Lab (Oh and Irvine, 2008)	N/A
Anti-βGal (Mouse) (1:200)	Developmental Studies Hybridoma Bank	JIE7
Anti-βGal (Chicken) (1:200)	Abcam	ab9361
Anti-Dcr2 (Rabbit) (1:800)	Abcam	ab4732
Anti-Diap1 (Rabbit) (1:200)	Bruce Hay	N/A
Anti-Notch, intracellular domain (Mouse) (1:250)	DSHB	C17.9c6
Anti-Vg (Rabbit) (1:100)	K. Guss (Guss Lab)	N/A
Anti-Atro (Rabbit) (1:500)	H. McNeill (McNeill Lab)(Yeung et al., 2017)	N/A
Anti-Atro (Mouse) (1:20)	Mattias Mannervik (Yeung et al., 2017)	N/A
Anti-E-cadherin (Rat) (1:200)	Developmental Studies Hybridoma Bank	DCAD2-c
Anti-Engrailed (Mouse) (1:10)	DSHB	4D9
Anti-FLAG (Mouse) (1:500) (IF S2 cells)	Sigma	Cat#F3165; RRID:AB_259529
Anti-FLAG (Mouse) (1:200) (IF tissue)	Sigma	Cat#F3165; RRID:AB_259529
Anti-FLAG (Mouse) (1:2000) (Western)	Sigma	Cat#F3165; RRID:AB_259529
Anti-FLAG (Rabbit) (1:500) (IF S2 cells)	Sigma	Cat#F7425; RRID:AB_439687
Anti-FLAG (Rabbit) (1:200) (IF tissue)	Sigma	Cat#F7425; RRID:AB_439687
Anti-FLAG (Rabbit) (1:2000) (Western)	Sigma	Cat#F7425; RRID:AB_439687
Anti-V5 (Mouse) (1:500) (IF S2 cells)	Invitrogen	Cat#R960-25; RRID:AB_2556564
Anti-V5 (Mouse) (1:5000) (Western)	Invitrogen	Cat#R960-25; RRID:AB_2556564
Anti-V5 (Rabbit) (1:500) (IF S2 cells)	Bethyl Laboratories	Cat#A190-120A; RRID:AB_67586
Anti-V5 (Rabbit) (1:5000) (Western)	Bethyl Laboratories	Cat#A190-120A; RRID:AB_67586

Development • Supplementary information

Anti-c-myc (Rat) (1:200) (IF S2 cells)	BIO-RAD	Cat#MCA1929; RRID:AB_322203
Anti-c-myc (Rat) (1:1000) (Western)	BIO-RAD	Cat#MCA1929; RRID:AB_322203
Anti-myc (Mouse) (1:1000) (Western)	Invitrogen	Cat#MA1-980 RRID:AB_558470
Anti-GAPDH (Mouse) (1:20,000) (Western)	Proteintech	Cat#60004-1-IG RRID:AB_2107436
Donkey anti-Mouse IgG (H+L) Alexa Fluor^{TM} 488 (1:100 IF tissue, 1:500 IF S2 cells)	Invitrogen	Cat#A-21202; RRID:AB_141607
Donkey anti-Rabbit IgG (H+L) Alexa Fluor^{TM} 488 (1:100 IF tissue, 1:500 IF S2 cells)	Invitrogen	Cat#A-21206; RRID:AB_2535792
Donkey anti-Chicken IgY (H+L) Alexa Fluor^{TM} 488 (1:100 IF tissue)	Invitrogen	Cat#A78948; RRID:AB_2921070
Cy-3AffiniPure Donkey anti-Rabbit IgG (H+L) (1:100)	Jackson Immuno Research Labs	Cat#711-165-152; RRID:AB_2307443
Cy3-AffiniPure Donkey Anti-Mouse IgG (H+L) (1:100)	Jackson Immuno Research Labs	Cat#715-165-151; RRID:AB_2315777
Cy3-AffiniPure Donkey Anti-Rat (1:100)	VWR	Cat#102649-822
Donkey anti-Rabbit IgG (H+L) Alexa Fluor 647-AffiniPure (1:100 IF tissue, 1:500 IF S2 cells)	Jackson Immuno Research Labs	Cat#711-605-152; RRID:AB_2492288
Donkey anti-Mouse IgG (H+L) Alexa Fluor 647-AffiniPure (1:100 IF tissue, 1:500 IF S2 cells)	Jackson Immuno Research Labs	Cat#715-605-151; RRID:AB_2340863
Donkey-anti-Rat 647 (1:100 IF tissue, 1:500 IF S2 cells)	Invitrogen	Cat#715-165-153 RRID: AB_2340694
IRDye 800CW Donkey anti-Rabbit IgG (1:20,000) (Western)	LICORbio	Cat#926-32213 RRID:AB_621848

Development • Supplementary information

IRDye 680RD Donkey anti-Mouse IgG (1:20,000) (Western)	LICORbio	Cat#926-68072 RRID:AB_10953628
Anti-Delta (Mouse) (1:200)	DSHB	C594.9B
Anti-Serrate (Rat) (1:200)	Irvine Lab (Papayannopoulos et al., 1998)	N/A

Development • Supplementary information
