# Supplementary material for: Regulation of Hippo signaling by Atrophin in the developing Drosophila wing
Source: Development. 2026 Aug 5;153(15):dev205540. doi: 10.1242/dev.205540 (PMC13480958; doi:10.1242/dev.205540)
Supplement: Supplementary file 2 [file develop-153-205540_review_history.pdf]

Development

Review COMMONS

Regulation of Hippo signaling by Atrophin in the developing Drosophila wing

Deimante Mikalauskaite, Cordelia Rauskolb, Tom Lehan, Srividya Venkatramanan, Lilia Carnes and Kenneth D. Irvine
DOI: 10.1242/dev.205540

Editor: James Briscoe

Review timeline

Submission to Review Commons:	8 October 2025
Submission to Development:	28 January 2026
Editorial decision:	28 January 2026
First revision received:	24 May 2026
Editorial decision:	28 June 2026
Second revision received:	30 June 2026
Accepted:	1 July 2026

Reviewer 1

Evidence, reproducibility and clarity

Summary

Atrophin (Atro) is a transcriptional co-repressor that is known to repress Fj expression, and associate with Trl, a co-factor of Yki. This manuscript explores the function of Atro in Hippo target gene expression.
Previous studies showed that Fj is also activates by Vg, and Ds is repressed by Vg. Here they show that the effect of Atro RNAi on Yki target gene expression depends on position. Loss of Atro leads to increased Yki target gene expression (Ex, Diap1)in distal cells near the DV boundary, and paradoxical decreased expression in more proximal regions. Interestingly, Vg and Atro can co-IP, suggesting they may act together to regulate gene expression, and overexpressed Sd can compete out Atro-Vg binding. Convincing data is also presented that loss of Atro leads to increased expression of Ds. These are strengths of the study. Weaknesses lie in the some of the figures (described below), the clarity of manuscript, and the linking of the biochemistry of Atro-Vg with the in vivo data.

Major Comments

Fig 3 shows very mild phenotypes, that are difficult to see, and could be made clearer with clonal expression of Atro-RNAi and UAS-Atro

Fig 3B. The cross sectional does not convincingly show differences in Yki aligning with the RFP signal . The biological significance of the very subtle changes in nuclear Yki is not clear

Fig 4 is unclear, and does not contribute much to the manuscript in its current state. This could be moved to supplemental data. Clones of Atro RNAi might help. Why is there a band of high Dachs GFP at the border of the clone in Fig 4D?

Fig 5E showing changes in Ex-lacZ is unconvincing, and at least requires quantification, and again

© 2026. Published by The Company of Biologists under the terms of the Creative Commons Attribution License (https://creativecommons.org/licenses/by/4.0/).

would be better done in clones.

Fig 6 D - the repression of Ds by Vg is not simple, and does not correlate in all clones- some places there is loss of Ds without an obvious Vg overexpressing clone, and in other places Vg overexpressing clones do not affect Ds expression. There are borders and fold that complicate the images. This should be addressed in the text.

Fig 7C-J do not add more information than reflecting the overexpression co-IP

The authors do not evenly describe the differing hypotheses in the discussion. The finding that they cannot detect a trimeric complex of Sd-Vg-Yki in overexpression S2 experiments does not preclude the possibility of complexes forming on chromatin. They briefly say they do not favor the idea that the increased expression of Yki target genes near the DV boundary is due to Atro repressive functions. However they do not directly test this. The finding that Sbb, a transcriptional co-repressor shows the same effects as Atro near the DV boundary is supportive of this idea. The discussion of why they favor the hypothesis that Atro regulation of Notch is responsible for the derepression of Yki targets is unclear (see final paragraph on P13). Do known co-factors such as G9a and HDAC similarly regulate Yki targets and Notch?

Minor comments

The text is unclear in several places. For example
P7. "Conversely, the negative influence of Atro on expression of Yki target genes in distal cells is independent of effects on Hippo signaling". This statement comes directly after the observation of subtle changes in Yki localization in Atro RNAi conditions. Since Atro is a transcriptional co-repressor, this seems an oversimplification at least.
Fig 1-- there is a double set of asteriks in 1F
Fig 2A is not critical and should be in supplemental

Significance

This manuscript is useful in pointing out that the effects of Atrophin on target gene expression in the Drosophila wing, specifically with regard to Yki target genes. The finding that loss of Atro leads to upregulation of Ds is interesting and novel, to a specialized audience. The finding that Atrophin binds to Vg and that increasing Sd blocks this interaction is novel and interesting. However the biochemistry is not well linked to the biology.

Reviewer 2

Evidence, reproducibility and clarity

Summary:

The authors describe a connection between Atrophin (encoded by grunge) and Hippo signaling. The authors perform their investigations in the wing imaginal disc, the precursor to the adult wing. The investigators manipulate (increase and decrease via RNAi) the expression of Atrophin (and other gene products) via the GAL4/UAS system in Drosophila. They used GAL4 drivers, usually engrailed-GAL4, that drive in the posterior compartment of the wing imaginal disc, so that nuanced responses in regions of this tissue that contribute to different features of the adult wing, including the notum, dorsal and ventral regions of the wing blade, and wing margin, can be uncovered, and compared to wild type events in the anterior wing compartment.
Through the experiments described in the manuscript, the authors connect, in the proximal regions of the wing pouch, that Atrophin and Vestigial together inhibit Dachsous. As a result of inhibition of Dachsous, Dachs remains active. This pathway member, in turn, inhibits Warts. When Warts is inhibited, Yorkie is localized to the nucleus, ultimately activating expression of target genes such as expanded and DIAP1. The authors show that Atrophin and Scalloped compete for Vestigial binding through different regions of the Vestigial protein, and that Scalloped disrupts colocalization of Vestigial and Atrophin.
The authors show that events are different at the wing margin, due to presence of Notch

© 2026. Published by The Company of Biologists under the terms of the Creative Commons Attribution License (https://creativecommons.org/licenses/by/4.0/).

signaling, but again involve Vestigial. The authors show that reduction of Atrophin at the margin results in reduced Vestigial. As a result, they argue, there is less Vestigial protein to compete with Yorkie for interaction with Scalloped, resulting in the higher expression of Yorkie target genes along the wing margin.

Major comments

The claims and comments are supported by the data. Data and methods support reproducibility. Experiments are adequately replicated and statistical analysis is adequate, with one exception. Would it be possible to provide quantitive data regarding the impact of Atrophin on Vestigial (protein and boundary element-lacZ) for figure 2, as was in figures 1, 3, and 4? This depiction of data would further illuminate the authors conclusions about Vestigial.

Minor comments:

In figures, it may be helpful to include notation such as A', A'', A''' to currently unidentified panels in figures. Is it possible to move some of the data in supplemental data to the main paper? For example, Figure S4 seems important to authors' conclusions so seems as if it should be in main body?

Significance

General assessment

The authors' approaches and models in Figure 1 and 8 allow readers to follow the different outcomes in the different regions of the wing disc. Experimental approaches are sound. The statistical analysis provided is helpful.

The contribution to the field is mechanistic.

To my knowledge, this study is the first to connect Atrophin and Yorkie. There are few references connecting Atrophin and Fat. Based on my cursory PubMed search, this manuscript describes the first direct link between Atrophin and yorkie in Drosophila. There is a vertebrate ref, Adler et al., 2013 PNAS, but I didn't see anything in Drosophila.

This study will be of most interest to those who investigate the mechanism(s) of Hippo signaling. Investigators who study Hippo signaling in other tissues and contexts in Drosophila may integrate study of Atrophin, and those who study this pathway in vertebrates may integrate the study of the vertebrate homolog RERE. The interest group would be focused, specialized.

My scientific area of expertise is Drosophila, wing imaginal disc, embryo, gene expression. I have not performed co immunoprecipitation studies as listed, but I know enough to follow them.

Reviewer 3

Evidence, reproducibility and clarity

Summary:

The study re-examines the role of the transcriptional co-repressor Atrophin in the wing imaginal disc in Drosophila, and especially its ability to regulate expression of Hippo pathway targets. The study confirms and extends previous work showing effects on Notch signaling near the tissue's distal, dorso-ventral boundary. The work also outlines opposite effects on Hippo signaling in proximal parts of the tissue and, for the first time, effects on regulators of the Fat pathway, which acts upsteam of Hippo in growth control. The authors provide both genetic and in vitro evidence that this latter effect may by via competition between newly discovered binding between Atrophin and Vestigial, which may reduce binding between Vg and the TEAD transcription factor Sd.

© 2026. Published by The Company of Biologists under the terms of the Creative Commons Attribution License (https://creativecommons.org/licenses/by/4.0/).

Major comments:

- Atrophin likely has several different partners and effects, complicating both past and the current genetic and biochemical analyses. The authors are thus appropriately cautious in their interpretations. They also do a good job describing previous work on Atrophin, and how their work confirms, extends, and in one case contradicts that work.
- The contradiction is that a previous study (Wang et al.) found that Atrophin knockdown reduced not only Notch activity but expression of a Notch transcriptional reporter. In contrast, this manuscript sees no effect on anti-Notch staining. The authors thus question the accuracy of the Notch reporter, but did not examine transcription directly.

Another possible cause of the discrepancy is different levels of knockdown. Have the authors tried used the same gal4 driver and RNAi line as Wang et al.? And in another study (Yeung et al.) Atrophin mutant clones appear to have (stronger?) effects on gene expression not noted in this manuscript.

Finally, are there any effects on Notch ligands?
- The authors show that Atrophin can bind the Sd cofactor Vg in vitro, and can thus inhibit Vg-Sd binding. Their model for activity in vivo is therefore based on changing the activity of the Vg-Sd complex. However, Yki also binds Sd. Thus it is possible that Atrophin, by freeing Sd from Vg, is making more Sd available for Yki; this would also increase Yki activity independently of any effects of Ds or Dachs levels. I am curious whether the authors have any data that bear on this? Even without data, a short discussion would be helpful.

Significance

The authors provide novel data on Atrophin activity, new possible targets and effectors of Atrophin, and a novel mechanism of action via binding to Vg. The in vivo genetic evidence is consistent with activity through Notch and Vg, although like most genetic evidence leaves open the possibility of parallel pathways. Given the importance of Drosophila and especially the imaginal discs for the examination of Hippo, Fat-Dachsous, and Notch signaling, this is a valuable contribution.

Author response to reviewers' comments

Preliminary Response to Reviewer’s Comments

We thank all three reviewers for their thoughtful and detailed comments, which have helped us to improve the quality and clarity of our manuscript.

Reviewer #1 (Evidence, reproducibility and clarity (Required)):

Summary
Atrophin (Atro) is a transcriptional co-repressor that is known to repress Fj expression, and associate with Trl, a co-factor of Yki. This manuscript explores the function of Atro in Hippo target gene expression.
Previous studies showed that Fj is also activates by Vg, and Ds is repressed by Vg. Here they show that the effect of Atro RNAi on Yki target gene expression depends on position. Loss of Atro leads to increased Yki target gene expression (Ex, Diap1) in distal cells near the DV boundary, and paradoxical decreased expression in more proximal regions. Interestingly, Vg and Atro can co-IP, suggesting they may act together to regulate gene expression, and overexpressed Sd can compete out Atro-Vg binding. Convincing data is also presented that loss of Atro leads to increased expression of Ds. These are strengths of the study. Weaknesses lie in the some of the figures (described below), the clarity of manuscript, and the linking of the biochemistry of Atro-Vg with the in vivo data.

Major Comments

© 2026. Published by The Company of Biologists under the terms of the Creative Commons Attribution License (https://creativecommons.org/licenses/by/4.0/).

-Fig 3 shows very mild phenotypes, that are difficult to see, and could be made clearer with clonal expression of Atro-RNAi and UAS-Atro

Response: We agree that the changes in nuclear Yki are mild, but they are nonetheless visible and reproducible, and we have quantified them. We do not think that the effects are more visible in clones. Indeed, we previously generated Atro over expression clones to examine effects on Yki subcellular localization but found that this approach did not improve visualization of changes in Yki localization, likely because the phenotype is mild and there is some variation in nuclear Yki levels from cell to cell.
Thus, a better estimation of the effect is obtained by examination over a larger area, as we do with expression of a posterior compartment Gal4. As noted below we will also try to obtain additional images to more clearly document the effect.

-Fig 3B. The cross sectional does not convincingly show differences in Yki aligning with the RFP signal. The biological significance of the very subtle changes in nuclear Yki is not clear

Response: We agree that the alignment of differences in Yki localization with the RFP signal is not very clear in this image. This might be because we used *en-Gal4*, which drives a late, weak expression on the anterior side of the A-P boundary, complicating analysis. To address this, we will repeat this experiment using a different Gal4 driver, *hh-Gal4*, that does not suffer from this issue.

Although changes in Yki localization appear subtle, they are sufficient to alter expression of Yki target genes, as illustrated in Fig 1. We also note that prior studies have shown that even mild changes in Yki localization detectable by antibody staining are associated with significant changes in expression of Yki target gene expression, and the differences in Yki that we observe are in line with these (eg see Rauskolb et al, Zyxin links fat signaling to the hippo pathway. *PLoS Biology* **9**, e1000624 (2011) or Alégot, H. *et al*. Recruitment of Jub by α-catenin promotes Yki activity and Drosophila wing growth. *Journal of Cell Science* **132**, jcs222018 (2019)).

-Fig 4 is unclear, and does not contribute much to the manuscript in its current state. This could be moved to supplemental data. Clones of Atro RNAi might help. Why is there a band of high Dachs GFP at the border of the clone in Fig 4D?

Response: We respectfully disagree, as we consider the data in Figure 4 a key result of our study. Fig. 4 demonstrates that Atro RNAi affects levels of Dachs, which is the central mediator of Ds-Fat signaling, and thus indicates that Ds-Fat signaling is being affected by Atro RNAi. Given the role of Dachs in mediating Ds-Fat regulation of Hippo signaling, the reduction of junctional Dachs reported in this figure could in principle be sufficient how Atro RNAi reduces Yki activity. We will revise text to try to make significance of this result clearer.

The band of higher Dachs:GFP at the AP compartment boundary in Fig 4D is consistent with our observations that levels of Fj are increased by Atro over-expression, as published observations have revealed that junctional Dachs is elevated along borders of Fj expression. We will revise the text to comment on this.

-Fig 5E showing changes in Ex-lacZ is unconvincing, and at least requires quantification, and again would be better done in clones.

Response: We agree the effect is weak and have now quantified it as suggested (quantitation in a new Fig 5F). We found that the modest difference in ex-lacZ between anterior and posterior cells in this Ds over-expression experiment was statistically significant in ventral cells but not in dorsal cells. The take home message from this analysis, that increases in Ds could contribute to but not fully explain the effect of Atro on Dachs levels and Yki activity, remains unchanged. We will also revise the text to make sure the interpretation of this result is clear. We don't agree that analyzing clones would make the results more clear; if anything it would complicate them as there are strong boundary effects associated with altered Ds expression (eg see Rogulja et al. Morphogen control of wing growth through the Fat signaling pathway. Developmental Cell 15, 309-321 (2008).)

-Fig 6 D - the repression of Ds by Vg is not simple, and does not correlate in all clones- some

© 2026. Published by The Company of Biologists under the terms of the Creative Commons Attribution License (https://creativecommons.org/licenses/by/4.0/).

places there is loss of Ds without an obvious Vg overexpressing clone, and in other places Vg overexpressing clones do not affect Ds expression. There are borders and fold that complicate the images. This should be addressed in the text.

Response: We appreciate the reviewers concern with this image, and there are a couple of complicating issues, including: 1) Ds expression is already quite low in the center of the wing pouch, which can make it hard to see a repression here (indeed Ds is likely repressed here by endogenous Vg), 2) the marker for the clones is visible in more basal focal planes from Ds protein, which can cause problems with alignment when the tissue is not flat. We have now replaced the Fig 6D image with a new example that shows the repression more clearly. We have also revised the text to discuss the issues raised.

-Fig 7C-J do not add more information than reflecting the overexpression co-IP

Response: While we agree that the results of the images in Fig 7C-J lead to the same conclusion as that from the co-IP experiments, we think that they are nonetheless still worth including as an independent experimental approach supporting the same conclusion.

-The authors do not evenly describe the differing hypotheses in the discussion. The finding that they cannot detect a trimeric complex of Sd-Vg-Yki in overexpression S2 experiments does not preclude the possibility of complexes forming on chromatin. They briefly say they do not favor the idea that the increased expression of Yki target genes near the DV boundary is due to Atro repressive functions. However they do not directly test this. The finding that Sbb, a transcriptional co-repressor shows the same effects as Atro near the DV boundary is supportive of this idea. The discussion of why they favor the hypothesis that Atro regulation of Notch is responsible for the derepression of Yki targets is unclear (see final paragraph on P13). Do known co-factors such as G9a and HDAC similarly regulate Yki targets and Notch?

Response: In regard to the hypothetical possibility of a trimeric complex of Sd-Vg-Atro forming on chromatin, we agree that we cannot exclude this, but the fact that we could not detect this complex in solution, and indeed found that Sd and Atro compete for binding to Vg, argues against it. Nonetheless we can mention it as a hypothetical possibility in the Discussion.

In regard to the possibility that Atro directly represses Yki target genes at the DV boundary. The primary reason that we do not favor this is that we think the effects of Atro here can be accounted for by the effects of Atro on Notch signaling that we document in the manuscript. We further note that we did in fact conduct transcriptional reporter assays in S2 cells to investigate whether we could find evidence that Atro could directly repress Yki targets, but we saw no effect. We didn’t include these studies in the manuscript as it’s a negative result, and we don’t have a good positive control for these experiments (ie a transcriptional reporter that we know is directly repressed by Atro in S2 cells).

The finding that Atro and Sbb RNAi have similar effects supports the idea that Atro is acting as a transcriptional repressor, but doesn’t tell us what the relevant direct target of repression is. The Co-factors G9a and HDAC have been shown previously to affect Notch target genes similarly to Atro (Wang et al., 2018, DOI: 10.1242/bio.029637), and Notch signaling was reported to affect Yki activity at the DV boundary of the wing in Djiane et al 2014.

We sincerely appreciate the Reviewer highlighting aspects of the Discussion that could be improved to provide a fuller explanation of our interpretation of the results obtained, as well as possible alternatives, and we will revise the Discussion accordingly.

Minor comments

-The text is unclear in several places. For example P7. "Conversely, the negative influence of Atro on expression of Yki target genes in distal cells is independent of effects on Hippo signaling". This statement comes directly after the observation of subtle changes in Yki localization in Atro RNAi conditions. Since Atro is a transcriptional co-repressor, this seems an oversimplification at least.

Response: We have revised the text to clarify these comments. The point here is that near the

© 2026. Published by The Company of Biologists under the terms of the Creative Commons Attribution License (https://creativecommons.org/licenses/by/4.0/).

DV boundary loss of Atro leads to decreased nuclear Yki, but upregulation of Yki target genes. We think the opposing effects on Yki vs Yki target genes are explained by the fact that Atro is affecting Yki target genes indirectly here, through regulation of Notch signaling.

-Fig 1-- there is a double set of asteriks in 1F

Response: Thank you for catching this, we will remove the additional asterisk.

-Fig 2A is not critical and should be in supplemental

Response: We would prefer to keep Fig, 2A as originally placed, because it's logically connected to the other results shown in this figure examining Notch target genes, this Notch staining image presents novel results relevant to understanding how Atro affects Notch signaling, and it's important to addressing issues raised by Reviewer 3.

Reviewer #1 (Significance (Required)):

This manuscript is useful in pointing out that the effects of Atrophin on target gene expression in the Drosophila wing, specifically with regard to Yki target genes. The finding that loss of Atro leads to upregulation of Ds is interesting and novel, to a specialized audience. The finding that Atrophin binds to Vg and that increasing Sd blocks this interaction is novel and interesting. However the biochemistry is not well linked to the biology.

Response: We thank the reviewer for their assessment and will make revisions to the text to address the concerns raised above and clarify our interpretations of the results.

Reviewer #2 (Evidence, reproducibility and clarity (Required)):

Summary:

The authors describe a connection between Atrophin (encoded by grunge) and Hippo signaling. The authors perform their investigations in the wing imaginal disc, the precursor to the adult wing. The investigators manipulate (increase and decrease via RNAi) the expression of Atrophin (and other gene products) via the GAL4/UAS system in Drosophila. They used GAL4 drivers, usually engrailed-GAL4, that drive in the posterior compartment of the wing imaginal disc, so that nuanced responses in regions of this tissue that contribute to different features of the adult wing, including the notum, dorsal and ventral regions of the wing blade, and wing margin, can be uncovered, and compared to wild type events in the anterior wing compartment.
Through the experiments described in the manuscript, the authors connect, in the proximal regions of the wing pouch, that Atrophin and Vestigial together inhibit Dachsous. As a result of inhibition of Dachsous, Dachs remains active. This pathway member, in turn, inhibits Warts. When Warts is inhibited, Yorkie is localized to the nucleus, ultimately activating expression of target genes such as expanded and DIAP1. The authors show that Atrophin and Scalloped compete for Vestigial binding through different regions of the Vestigial protein, and that Scalloped disrupts colocalization of Vestigial and Atrophin.
The authors show that events are different at the wing margin, due to presence of Notch signaling, but again involve Vestigial. The authors show that reduction of Atrophin at the margin results in reduced Vestigial. As a result, they argue, there is less Vestigial protein to compete with Yorkie for interaction with Scalloped, resulting in the higher expression of Yorkie target genes along the wing margin.

Major comments

-The claims and comments are supported by the data. Data and methods support reproducibility. Experiments are adequately replicated and statistical analysis is adequate, with one exception. Would it be possible to provide quantitive data regarding the impact of Atrophin on Vestigial (protein and boundary element-lacZ) for figure 2, as was in figures 1, 3, and 4? This depiction of data would further illuminate the authors conclusions about Vestigial.

© 2026. Published by The Company of Biologists under the terms of the Creative Commons Attribution License (https://creativecommons.org/licenses/by/4.0/).

Response: We will include the requested quantitation. For the *vestigial* boundary enhancer, we have completed the quantitation and observed significant effects. Quantitation of Vestigial protein expression will also be performed and included, and we will also modify the text to discuss the results of the quantitation.

Minor comments:

-In figures, it may be helpful to include notation such as A', A'', A''' to currently unidentified panels in figures. Is it possible to move some of the data in supplemental data to the main paper? For example, Figure S4 seems important to authors' conclusions so seems as if it should be in main body?

Response: We have now added the suggested additional notations to the figure panels.

Fig S4 maps regions of Vg that associate with Atro. We have moved it to the main figures, as requested.

Reviewer #2 (Significance (Required)):

General assessment

The authors' approaches and models in Figure 1 and 8 allow readers to follow the different outcomes in the different regions of the wing disc. Experimental approaches are sound. The statistical analysis provided is helpful.

The contribution to the field is mechanistic.

To my knowledge, this study is the first to connect Atrophin and Yorkie. There are few references connecting Atrophin and Fat. Based on my cursory PubMed search, this manuscript describes the first direct link between Atrophin and yorkie in Drosophila. There is a vertebrate ref, Adler et al., 2013 PNAS, but I didn't see anything in Drosophila.

This study will be of most interest to those who investigate the mechanism(s) of Hippo signaling. Investigators who study Hippo signaling in other tissues and contexts in Drosophila may integrate study of Atrophin, and those who study this pathway in vertebrates may integrate the study of the vertebrate homolog RERE. The interest group would be focused, specialized.

My scientific area of expertise is Drosophila, wing imaginal disc, embryo, gene expression. I have not performed co immunoprecipitation studies as listed, but I know enough to follow them.

Response: We thank the reviewer for these comments. We note also that the Adler et al 2013 PNAS Reference identifies functional connection between YAP (mammalian Yki) and a protein called “atrophin-1 interacting protein 4” (AIP4) but does not actually describe any connection to Atrophin.

Reviewer #3 (Evidence, reproducibility and clarity (Required)):

Summary:
The study re-examines the role of the transcriptional co-repressor Atrophin in the wing imaginal disc in Drosophila, and especially its ability to regulate expression of Hippo pathway targets. The study confirms and extends previous work showing effects on Notch signaling near the tissue's distal, dorso-ventral boundary. The work also outlines opposite effects on Hippo signaling in proximal parts of the tissue and, for the first time, effects on regulators of the Fat pathway, which acts upsteam of Hippo in growth control. The authors provide both genetic and in vitro evidence that this latter effect may by via competition between newly discovered binding between Atrophin and Vestigial, which may reduce binding between Vg and the TEAD transcription factor Sd.

Major comments:

© 2026. Published by The Company of Biologists under the terms of the Creative Commons Attribution License (https://creativecommons.org/licenses/by/4.0/).

• Atrophin likely has several different partners and effects, complicating both past and the current genetic and biochemical analyses. The authors are thus appropriately cautious in their interpretations. They also do a good job describing previous work on Atrophin, and how their work confirms, extends, and in one case contradicts that work.

Response: Thank-you

• The contradiction is that a previous study (Wang et al.) found that Atrophin knockdown reduced not only Notch activity but expression of a Notch transcriptional reporter. In contrast, this manuscript sees no effect on anti-Notch staining. The authors thus question the accuracy of the Notch reporter, but did not examine transcription directly.

Response: We consider the effect on Notch protein levels to ultimately be more relevant to whether changes in Notch levels could explain effects on Notch target genes than whether a *Notch-lacZ* reporter line is affected. Additionally, as noted the expression pattern of the *Notch-lacZ* line used in Wang et al. does not match endogenous Notch protein expression. For these reasons we do not think it is worthwhile for us to examine the *Notch-lacZ* line.

Another possible cause of the discrepancy is different levels of knockdown. Have the authors tried used the same gal4 driver and RNAi line as Wang et al.? And in another study (Yeung et al.) Atrophin mutant clones appear to have (stronger?) effects on gene expression not noted in this manuscript.

Response: Both our experiments and results reported in Wang et al., 2018 used *en- Gal4,* and we also used the same Atro RNAi fly line, so this is unlikely to explain the difference in our conclusions. Yeung et al examined two Notch targets in the wing discs, a N signaling reporter (NRE-GFP) and Cut. Wang et al 2018 reported effects on Wg and Cut. We reported the effect on Vg and the Vg boundary enhancer because it's relevant to our study of how Atro affects Yki target genes. We have in fact also observed effects on Wg and Cut similar to those reported in the earlier studies but we did not include these results as we didn't think it made sense for us to include a repetition of already published results. We agree that Atro mutant clones could potentially have even stronger effects, but unfortunately we found that the previously described fly line with an Atro null allele on an FRT chromosome was no longer mutant for Atro, so it wasn't a feasible experiment. Finally, we emphasize that the strong knockdown of Atro achieved in our RNAi experiments is documented in Fig S1 and it is sufficient to have reproducible effects on several different genes that we examined.

Finally, are there any effects on Notch ligands?

Response: We plan to address this by examining expression of Ser and Dl protein in wing discs with Atro knockdown. Based on prior studies that have established that Notch ligands are positive feedback targets on Notch signaling in the wing disc, we expect that there likely will be some effect on their expression.

• The authors show that Atrophin can bind the Sd cofactor Vg in vitro, and can thus inhibit Vg-Sd binding. Their model for activity in vivo is therefore based on changing the activity of the Vg-Sd complex. However, Yki also binds Sd. Thus it is possible that Atrophin, by freeing Sd from Vg, is making more Sd available for Yki; this would also increase Yki activity independently of any effects of Ds or Dachs levels. I am curious whether the authors have any data that bear on this? Even without data, a short discussion would be helpful.

Response: This is an interesting point. Simply over-expressing Sd generally does not lead to more Yki activity; it actually (at least at high levels) induces cell death. This is likely because Sd is not limiting for Sd-Yki activity, nuclear Yki is. Conversely, when Sd levels are increased it could potentially interact with Tgi, repressing rather than activating expression of Sd-Yki target genes. The actual effect observed thus will depend on how much extra Sd is available, and on the relative nuclear levels of available partners including Yki and Tgi. While we can't be certain whether there is any impact of Sd potentially released from Vg under higher Atro levels, what we can say is that we have clear effects on expression of genes regulated by Vg (eg Ds and Fj) and we think this can explain the results obtained. We will nonetheless discuss the point raised here by the Reviewer in

© 2026. Published by The Company of Biologists under the terms of the Creative Commons Attribution License (https://creativecommons.org/licenses/by/4.0/).

the revised manuscript.

Reviewer #3 (Significance (Required)):

The authors provide novel data on Atrophin activity, new possible targets and effectors of Atrophin, and a novel mechanism of action via binding to Vg. The in vivo genetic evidence is consistent with activity through Notch and Vg, although like most genetic evidence leaves open the possibility of parallel pathways. Given the importance of Drosophila and especially the imaginal discs for the examination of Hippo, Fat- Dachsous, and Notch signaling, this is a valuable contribution.

Response: We thank the reviewer for their comments.

Original submission

First decision letter

MS ID#: dev.205540

MS Title: Regulation of Hippo signaling by Atrophin in the developing Drosophila wing

Authors: Deimante Mikalauskaite; Cordelia Rauskolb; Tom Lehan; Srividya Venkatramanan; Lilia Carnes; Kenneth D. Irvine

Dear Dr Irvine,

Thank you for sending your manuscript to Development through Review Commons.

Many thanks for transferring your manuscript to Development from Review Commons. I have now had the chance to review your documents, and we would like to invite you to revise your manuscript according to your revision plan. Once we receive the revised version, we may seek further input from the Review Commons referees. If you have any questions about this process, please do get in touch.

First revision

Author response to reviewers' comments

We thank all three reviewers for their thoughtful and detailed comments, which have helped us to improve the quality and clarity of our manuscript.

Reviewer #1 (Evidence, reproducibility and clarity (Required)):

Summary

Atrophin (Atro) is a transcriptional co-repressor that is known to repress Fj expression, and associate with Trl, a co-factor of Yki. This manuscript explores the function of Atro in Hippo target gene expression.

Previous studies showed that Fj is also activates by Vg, and Ds is repressed by Vg. Here they show that the effect of Atro RNAi on Yki target gene expression depends on position. Loss of Atro leads to increased Yki target gene expression (Ex, Diap1) in distal cells near the DV boundary, and paradoxical decreased expression in more proximal regions. Interestingly, Vg and Atro can co-IP,

© 2026. Published by The Company of Biologists under the terms of the Creative Commons Attribution License (https://creativecommons.org/licenses/by/4.0/).

suggesting they may act together to regulate gene expression, and overexpressed Sd can compete out Atro-Vg binding. Convincing data is also presented that loss of Atro leads to increased expression of Ds. These are strengths of the study. Weaknesses lie in the some of the figures (described below), the clarity of manuscript, and the linking of the biochemistry of Atro-Vg with the in vivo data.

Major Comments

-Fig 3 shows very mild phenotypes, that are difficult to see, and could be made clearer with clonal expression of Atro-RNAi and UAS-Atro

Response: We agree that the changes in nuclear Yki are mild, but they are nonetheless visible and reproducible, and we have quantified them. We do not think that the effects are more visible in clones. Indeed, we previously generated Atro over expression clones to examine effects on Yki subcellular localization but found that this approach did not improve visualization of changes in Yki localization, likely because the phenotype is mild and there is some variation in nuclear Yki levels from cell to cell. Thus, a better estimation of the effect is obtained by examination over a larger area, as we do with expression of a posterior compartment Gal4. What we have done instead to address this is to repeat the analysis with hh-Gal4, instead of the en-Gal4 used in the original submitted manuscript. hh-Gal4 is a stronger driver than en-Gal4, and has a sharper boundary of expression, and consequently we think this makes the effects on Yki localization clearer.

-Fig 3B. The cross sectional does not convincingly show differences in Yki aligning with the RFP signal. The biological significance of the very subtle changes in nuclear Yki is not clear

Response: We agree that the alignment of differences in Yki localization with the RFP signal was not very clear in this image. This might be because we used *en-Gal4*, which drives a late, weak expression on the anterior side of the A-P boundary, complicating analysis. To address this, we repeated this experiment using a different Gal4 driver, *hh-Gal4*, which has a sharp boundary of expression and makes the differences clearer.

Although changes in Yki localization appear subtle, they are sufficient to alter expression of Yki target genes, as illustrated in Fig 1. We also note that prior studies have shown that even mild changes in Yki localization detectable by antibody staining are associated with visible changes in expression of Yki target gene expression and wing growth, and the differences in Yki that we observe are in line with these (eg see Alégot, H. *et al*. *Journal of Cell Science* 132, jcs222018 (2019)).

-Fig 4 is unclear, and does not contribute much to the manuscript in its current state. This could be moved to supplemental data. Clones of Atro RNAi might help. Why is there a band of high Dachs GFP at the border of the clone in Fig 4D?

Response: We respectfully disagree, as we consider the data in Figure 4 a key result of our study. Fig. 4 demonstrates that Atro RNAi affects levels of Dachs, which is the central mediator of Ds-Fat signaling, and thus indicates that Ds-Fat signaling is being affected by Atro RNAi. Given the key role of Dachs in mediating Ds-Fat regulation of Hippo signaling, the reduction of junctional Dachs reported in this figure could in principle be sufficient to explain how Atro RNAi reduces Yki activity. We revised the text to try to make significance of this result clearer.

The band of higher Dachs:GFP at the AP compartment boundary in Fig 4D is consistent with our observation that levels of Fj are increased by Atro over-expression (Fig S4), as published observations have revealed that junctional Dachs is elevated along borders of Fj expression (eg see Mao et al, *Development* 133, 2539-2551 2006).

-Fig 5E showing changes in Ex-lacZ is unconvincing, and at least requires quantification, and again would be better done in clones.

Response: We agree the effect is weak and have now quantified it as suggested (quantitation in a new Fig 5F). We found that the modest difference in ex-lacZ between anterior and posterior cells in this Ds over-expression experiment was statistically significant in ventral cells but not in dorsal cells. The take home message from this analysis, that increases in Ds could contribute to but not fully explain the effect of Atro on Dachs levels and Yki activity, remains unchanged. We have also

© 2026. Published by The Company of Biologists under the terms of the Creative Commons Attribution License (https://creativecommons.org/licenses/by/4.0/).

revised the text to make sure the interpretation of this result is clear. We don't agree that analyzing clones would make the results more clear; if anything it would complicate them as there are strong boundary effects associated with altered Ds expression (eg see Rogulja et al. *Developmental Cell* 15, 309–321 (2008).)

-Fig 6 D - the repression of Ds by Vg is not simple, and does not correlate in all clones- some places there is loss of Ds without an obvious Vg overexpressing clone, and in other places Vg overexpressing clones do not affect Ds expression. There are borders and fold that complicate the images. This should be addressed in the text.

Response: We appreciate the reviewers concern with this image, and there are a couple of complicating issues, including: 1) Ds expression is already quite low in the center of the wing pouch, which can make it hard to see a repression here (indeed Ds is presumably repressed here by endogenous Vg), 2) the marker for the clones is visible in more basal focal planes from Ds protein, which can cause problems with alignment when the tissue is not flat. We have now replaced the Fig 6D image with a new example that shows the repression more clearly. We have also revised the text to discuss this.

-Fig 7C-J do not add more information than reflecting the overexpression co-IP

Response: While we agree that the results of the images in Fig 7C-J lead to the same conclusion as that from the co-IP experiments, we think that they are nonetheless still worth including as an independent experimental approach supporting the same conclusion, and so we have kept it in the manuscript.

-The authors do not evenly describe the differing hypotheses in the discussion. The finding that they cannot detect a trimeric complex of Sd-Vg-Yki in overexpression S2 experiments does not preclude the possibility of complexes forming on chromatin. They briefly say they do not favor the idea that the increased expression of Yki target genes near the DV boundary is due to Atro repressive functions. However they do not directly test this. The finding that Sbb, a transcriptional co-repressor shows the same effects as Atro near the DV boundary is supportive of this idea. The discussion of why they favor the hypothesis that Atro regulation of Notch is responsible for the derepression of Yki targets is unclear (see final paragraph on P13). Do known co-factors such as G9a and HDAC similarly regulate Yki targets and Notch?

Response: In regard to the hypothetical possibility of a trimeric complex of Sd-Vg-Atro forming on chromatin, we agree that we cannot exclude this, but the fact that we could not detect this complex in solution, and instead found that Sd and Atro compete for binding to Vg, argues against it. Nonetheless we now mention it as a hypothetical possibility in the Discussion.

In regard to the possibility that Atro directly represses Yki target genes at the DV boundary. The primary reason that we do not favor this is that we think the effects of Atro here can be accounted for by the effects of Atro on Notch signaling that we document in the manuscript. We further note that we did in fact conduct transcriptional reporter assays in S2 cells to investigate whether we could find evidence that Atro could directly repress Yki targets (eg *ex*), but we saw no effect. We didn't include these studies in the manuscript as it's a negative result, and we don't have a good positive control for these experiments (ie a transcriptional reporter that we know is directly repressed by Atro in S2 cells).

The finding that Atro and Sbb RNAi have similar effects supports the idea that Atro is acting as a transcriptional repressor, but doesn't tell us what the relevant direct target of repression is. The Co-factors G9a and HDAC have been shown previously to affect Notch target genes similarly to Atro (Wang et al., 2018, DOI: 10.1242/bio.029637), and we now note this in the Discussion. Notch signaling was reported to affect Yki activity at the DV boundary of the wing in Djiane et al 2014.

We sincerely appreciate the Reviewer highlighting aspects of the Discussion that could be improved to provide a fuller explanation of our interpretation of the results obtained, as well as possible alternatives, and we have revised the Discussion accordingly.

Minor comments

© 2026. Published by The Company of Biologists under the terms of the Creative Commons Attribution License (https://creativecommons.org/licenses/by/4.0/).

-The text is unclear in several places. For example

P7. "Conversely, the negative influence of Atro on expression of Yki target genes in distal cells is independent of effects on Hippo signaling". This statement comes directly after the observation of subtle changes in Yki localization in Atro RNAi conditions. Since Atro is a transcriptional co-repressor, this seems an oversimplification at least.

Response: We have revised the text to clarify these comments. The point here is that near the DV boundary loss of Atro leads to decreased nuclear Yki, but upregulation of Yki target genes. We think the opposing effects on Yki vs Yki target genes are explained by the fact that Atro is affecting Yki target genes indirectly here, through regulation of Notch signaling.

-Fig 1-- there is a double set of asteriks in 1F

Response: Thank you for catching this, we have removed the additional asterisk.

-Fig 2A is not critical and should be in supplemental

Response: We prefer to keep Fig 2A as originally placed, because 1) it's logically connected to the other results shown in this figure examining Notch target genes, 2) this Notch staining image presents novel results relevant to understanding how Atro affects Notch signaling, and 3) it's relevant to addressing issues raised by Reviewer 3.

Reviewer #1 (Significance (Required)):

This manuscript is useful in pointing out that the effects of Atrophin on target gene expression in the Drosophila wing, specifically with regard to Yki target genes. The finding that loss of Atro leads to upregulation of Ds is interesting and novel, to a specialized audience. The finding that Atrophin binds to Vg and that increasing Sd blocks this interaction is novel and interesting. However the biochemistry is not well linked to the biology.

Response: We thank the reviewer for their assessment and have made revisions to address the concerns raised above and clarify our interpretations of the results.

Reviewer #2 (Evidence, reproducibility and clarity (Required)):

<u>Summary:</u>

The authors describe a connection between Atrophin (encoded by grunge) and Hippo signaling. The authors perform their investigations in the wing imaginal disc, the precursor to the adult wing. The investigators manipulate (increase and decrease via RNAi) the expression of Atrophin (and other gene products) via the GAL4/UAS system in Drosophila. They used GAL4 drivers, usually engrailed-GAL4, that drive in the posterior compartment of the wing imaginal disc, so that nuanced responses in regions of this tissue that contribute to different features of the adult wing, including the notum, dorsal and ventral regions of the wing blade, and wing margin, can be uncovered, and compared to wild type events in the anterior wing compartment.

Through the experiments described in the manuscript, the authors connect, in the proximal regions of the wing pouch, that Atrophin and Vestigial together inhibit Dachsous. As a result of inhibition of Dachsous, Dachs remains active. This pathway member, in turn, inhibits Warts. When Warts is inhibited, Yorkie is localized to the nucleus, ultimately activating expression of target genes such as expanded and DIAP1. The authors show that Atrophin and Scalloped compete for Vestigial binding through different regions of the Vestigial protein, and that Scalloped disrupts colocalization of Vestigial and Atrophin.

The authors show that events are different at the wing margin, due to presence of Notch signaling, but again involve Vestigial. The authors show that reduction of Atrophin at the margin results in reduced Vestigial. As a result, they argue, there is less Vestigial protein to compete with Yorkie for

© 2026. Published by The Company of Biologists under the terms of the Creative Commons Attribution License (https://creativecommons.org/licenses/by/4.0/).

interaction with Scalloped, resulting in the higher expression of Yorkie target genes along the wing margin.

Major comments

-The claims and comments are supported by the data. Data and methods support reproducibility. Experiments are adequately replicated and statistical analysis is adequate, with one exception. Would it be possible to provide quantitive data regarding the impact of Atrophin on Vestigial (protein and boundary element-lacZ) for figure 2, as was in figures 1, 3, and 4? This depiction of data would further illuminate the authors conclusions about Vestigial.

Response: We have now included the requested quantitation (Fig. 2E,I), which confirms decreased Vg and vg- boundary enhancer expression when Atro is reduced and increased expression when Atro is increased.

Minor comments:

-In figures, it may be helpful to include notation such as A', A", A"' to currently unidentified panels in figures. Is it possible to move some of the data in supplemental data to the main paper? For example, Figure S4 seems important to authors' conclusions so seems as if it should be in main body?

Response: We have now added the suggested additional notations to the figure panels.

Fig S4 maps regions of Vg that associate with Atro. We have moved it to the main figures (now Fig. 8), as requested.

Reviewer #2 (Significance (Required)):

General assessment

The authors' approaches and models in Figure 1 and 8 allow readers to follow the different outcomes in the different regions of the wing disc. Experimental approaches are sound. The statistical analysis provided is helpful.

The contribution to the field is mechanistic.

To my knowledge, this study is the first to connect Atrophin and Yorkie. There are few references connecting Atrophin and Fat. Based on my cursory PubMed search, this manuscript describes the first direct link between Atrophin and yorkie in Drosophila. There is a vertebrate ref, Adler et al., 2013 PNAS, but I didn't see anything in Drosophila.

This study will be of most interest to those who investigate the mechanism(s) of Hippo signaling. Investigators who study Hippo signaling in other tissues and contexts in Drosophila may integrate study of Atrophin, and those who study this pathway in vertebrates may integrate the study of the vertebrate homolog RERE. The interest group would be focused, specialized.

My scientific area of expertise is Drosophila, wing imaginal disc, embryo, gene expression. I have not performed co immunoprecipitation studies as listed, but I know enough to follow them.

Response: We thank the reviewer for these comments. We note also that the Adler et al 2013 PNAS Reference identifies a functional connection between YAP (mammalian Yki homolog) and a protein called “atrophin-1 interacting protein 4” (AIP4) but does not actually describe any connection to Atrophin.

Reviewer #3 (Evidence, reproducibility and clarity (Required)):

Summary:

© 2026. Published by The Company of Biologists under the terms of the Creative Commons Attribution License (https://creativecommons.org/licenses/by/4.0/).

The study re-examines the role of the transcriptional co-repressor Atrophin in the wing imaginal disc in Drosophila, and especially its ability to regulate expression of Hippo pathway targets. The study confirms and extends previous work showing effects on Notch signaling near the tissue's distal, dorso-ventral boundary. The work also outlines opposite effects on Hippo signaling in proximal parts of the tissue and, for the first time, effects on regulators of the Fat pathway, which acts upsteam of Hippo in growth control. The authors provide both genetic and in vitro evidence that this latter effect may by via competition between newly discovered binding between Atrophin and Vestigial, which may reduce binding between Vg and the TEAD transcription factor Sd.

Major comments:

• Atrophin likely has several different partners and effects, complicating both past and the current genetic and biochemical analyses. The authors are thus appropriately cautious in their interpretations. They also do a good job describing previous work on Atrophin, and how their work confirms, extends, and in one case contradicts that work.

Response: Thank-you

• The contradiction is that a previous study (Wang et al.) found that Atrophin knockdown reduced not only Notch activity but expression of a Notch transcriptional reporter. In contrast, this manuscript sees no effect on anti-Notch staining. The authors thus question the accuracy of the Notch reporter, but did not examine transcription directly.

Response: We consider the effect on Notch protein levels to ultimately be more relevant to whether changes in Notch levels could explain effects on Notch target genes than whether a *Notch-lacZ* reporter line is affected. Additionally, as noted the expression pattern of the *Notch-lacZ* line used in Wang et al. does not match endogenous Notch protein expression. For these reasons we do not think it is worthwhile for us to examine the *Notch-lacZ* line.

Another possible cause of the discrepancy is different levels of knockdown. Have the authors tried used the same gal4 driver and RNAi line as Wang et al.? And in another study (Yeung et al.) Atrophin mutant clones appear to have (stronger?) effects on gene expression not noted in this manuscript.

Response: Both our experiments and results reported in Wang et al., 2018 used *en-Gal4*, and we also used the same Atro RNAi fly line, so this is unlikely to explain the difference in our conclusions. Yeung et al examined two Notch targets in the wing discs, a N signaling reporter (NRE-GFP) and Cut. Wang et al 2018 reported effects on Wg and Cut. We reported the effect on Vg and the Vg boundary enhancer because it's relevant to our study of how Atro affects Yki target genes. We have in fact also observed effects on Wg and Cut similar to those reported in the earlier studies but we did not include these results as we didn't think it made sense for us to include a repetition of already published results. We agree that Atro mutant clones could potentially have even stronger effects, but unfortunately we found that the previously described fly line with an Atro null allele on an FRT chromosome was no longer mutant for Atro, so this wasn't a feasible experiment. Finally, we emphasize that the strong knockdown of Atro achieved in our RNAi experiments is documented in Fig S1 and it is sufficient to have reproducible effects on several different genes that we examined.

Finally, are there any effects on Notch ligands?

Response: We have addressed this by examining expression of Ser and Delta protein in wing discs with Atro knockdown or over-expression (new Fig S2). We observed little or no decrease in expression with Atro RNAi, and a broadening of ligand expression with Atro over-expression together with slight changes in the levels of expression at the DV boundary. Thus changes in Notch ligand expression cannot explain the changes in Notch activity at the DV boundary that we and others have observed.

• The authors show that Atrophin can bind the Sd cofactor Vg in vitro, and can thus inhibit Vg-Sd binding. Their model for activity in vivo is therefore based on changing the activity of the Vg-Sd

© 2026. Published by The Company of Biologists under the terms of the Creative Commons Attribution License (https://creativecommons.org/licenses/by/4.0/).

complex. However, Yki also binds Sd. Thus it is possible that Atrophin, by freeing Sd from Vg, is making more Sd available for Yki; this would also increase Yki activity independently of any effects of Ds or Dachs levels. I am curious whether the authors have any data that bear on this? Even without data, a short discussion would be helpful.

Response: This is an interesting point. Simply over-expressing Sd generally does not lead to more Yki activity; it actually (at least at high levels) induces cell death (eg see Liu et al Developmental Biology 228, 287-303, 2000). This is likely because Sd is not limiting for Sd-Yki activity, nuclear Yki is. Conversely, when Sd levels are increased it could potentially interact with Tgi, repressing rather than activating expression of Sd-Yki target genes. The actual effect observed thus will depend on how much extra Sd is available, and on the relative nuclear levels of available partners including Yki and Tgi. While we can't be certain whether there is any impact of Sd potentially released from Vg under higher Atro levels, what we can say is that we have clear effects on expression of genes regulated by Vg (eg Ds and Fj) and we think this can explain the results obtained. We now discuss the point raised here by the Reviewer in the revised manuscript.

Reviewer #3 (Significance (Required)):

The authors provide novel data on Atrophin activity, new possible targets and effectors of Atrophin, and a novel mechanism of action via binding to Vg. The in vivo genetic evidence is consistent with activity through Notch and Vg, although like most genetic evidence leaves open the possibility of parallel pathways. Given the importance of Drosophila and especially the imaginal discs for the examination of Hippo, Fat-Dachsous, and Notch signaling, this is a valuable contribution.

Response: We thank the reviewer for their comments.

Second decision letter

MS ID#: dev.205540R1

MS Title: Regulation of Hippo signaling by Atrophin in the developing Drosophila wing

Authors: Deimante Mikalauskaite; Cordelia Rauskolb; Tom Lehan; Srividya Venkatramanan; Lilia Carnes; Kenneth D. Irvine

Dear Dr Irvine,

I have now received all the referees reports on the above manuscript, and have reached a decision. The referees' comments are appended below.

The overall evaluation is positive and we would like to publish a revised manuscript in Development, provided that the referees' comments can be satisfactorily addressed. Reviewer 2 requests some clarifications and minor adjustments. Please attend to all of the reviewers' comments in your revised manuscript and detail them in your point-by-point response. If you do not agree with any of their criticisms or suggestions explain clearly why this is so. If it would be helpful, you are welcome to contact us to discuss your revision in greater detail. Please send us a point-by-point response indicating your plans for addressing the referees' comments, and we will look over this and provide further guidance.

Reviewer 2

SUMMARY OF THE ADVANCE MADE IN THIS PAPER AND ITS POTENTIAL SIGNIFICANCE TO THE FIELD

As noted previously, this study provides some important advances on the role of the complex transcriptional cofactor Atrophin, and provides evidence for novel routes by which it has opposite,

© 2026. Published by The Company of Biologists under the terms of the Creative Commons Attribution License (https://creativecommons.org/licenses/by/4.0/).

context-dependent effects on regulators and outputs of the Hippo growth-control pathway in Drosophila. While the manuscript's conclusions are at times speculative, the authors do a good job acknowledging this and discussion alternative. The evidence that Atrophin can bind Vg and reduce Vg-Sd binding, and thus Vg-Sd activity, is intriguing, as is the analyses of activity by Vg-Sd-dependent expression of Fat-Dachsous pathway members.

SUGGESTIONS TO AUTHORS

My new comments are minor, but should be considered when preparing the final manuscript.

1) Gal80ts- the authors use this to temporally gate manipulations to Atrophin activity, but only mention this, and the rationale behind it, in the methods. It would help the reader if that was included in the Results section.

2) " Similar effects were observed using an independent Atro RNAi line (Fig. S1F)." This reads a bit oddly as the paragraph never says which line was used for the other data shown. Maybe just say, as in the previous paragraph, that results were obtained with two different RNAis.

3) "When Yki levels were reduced in posterior wing cells by RNAi, the expression of Yki target genes was strongly reduced throughout the posterior compartment (Fig. 1K, Fig. S1G). When knockdown of Atro was combined with knockdown of Yki, the effect of Yki knockdown was partially reversed, as low levels of ex-lacZ and Diap1 were observed throughout the wing pouch (Fig. 1L, Fig. S1H)." This could be clearer, as the partial reversal seems to rest on the difference between "strongly reduced" and "low levels".

4)"was reduced near the center of the wing pouch" Do the authors mean distally? I'm not sure the term center is used previously except in the figure legend. Center can be a bit confusing, as it is a term that means different places in the wing pouch vs the wing blade.
Also, while were on terms for axes, it would help the reader to include a "proximal" tag on the dorsal side of the wing pouch as well as the ventral in Fig. 1B, more consistent with the white arrows used elsewhere in Fig. 1.

5) "As for Atro, this reduction in nuclear Yki was accompanied by increased levels of Ds and reduced junctional levels of Dachs (Fig. S5D,E)." The authors mean I think "As shown previously for Atro". As for often has a different meaning. As for Frank, he loved Trump's changes at the NIH.

Reviewer 3

SUMMARY OF THE ADVANCE MADE IN THIS PAPER AND ITS POTENTIAL SIGNIFICANCE TO THE FIELD

In this manuscript Mikalauskaite, et al. investigate the link between Atro and Hippo signaling in Drosophila wing discs. They show - by modulating Atro expression levels - that Atro regulates the expression of Hippo/Yki pathway target genes (diap1 and ex). They also show that modulating Atro levels can rescue the effects (at least partially) of increased or decreased Yki activity in the wing disc. They demonstrate that Atro regulates Hippo pathway signaling by promoting nuclear accumulation of Yki, and that Atro regulates Fj/Vg levels and Dachs localization at junctions. Thus, Atro regulates Hippo signaling by interacting with the Ft-Ds pathway. Lastly, the authors demonstrate that Atro physically binds to Vg, and that this binding likely interferes with Vg-Sd binding. In sum, Mikalauskaite, et al. show that Atro is a multifunctional transcriptional factor that integrates Notch signaling, Vg activity, and Ds-Ft-Hippo signaling to regulate Yki target gene expression. In its current state the manuscript is suitable for publication. The authors did a good job of addressing reviewer concerns/comments and incorporating reviewer recommendations.

SUGGESTIONS TO AUTHORS

Second revision

© 2026. Published by The Company of Biologists under the terms of the Creative Commons Attribution License (https://creativecommons.org/licenses/by/4.0/).

Author response to reviewers' comments

Response to Comments from the Reviewers:

Reviewer 2:

SUMMARY OF THE ADVANCE MADE IN THIS PAPER AND ITS POTENTIAL SIGNIFICANCE TO THE FIELD

As noted previously, this study provides some important advances on the role of the complex transcriptional cofactor Atrophin, and provides evidence for novel routes by which it has opposite, context-dependent effects on regulators and outputs of the Hippo growth-control pathway in Drosophila. While the manuscript's conclusions are at times speculative, the authors do a good job acknowledging this and discussion alternative. The evidence that Atrophin can bind Vg and reduce Vg-Sd binding, and thus Vg-Sd activity, is intriguing, as is the analyses of activity by Vg-Sd-dependent expression of Fat-Dachsous pathway members.

Response: Thank-you!

SUGGESTIONS TO AUTHORS

My new comments are minor, but should be considered when preparing the final manuscript.

1) Gal80ts- the authors use this to temporally gate manipulations to Atrophin activity, but only mention this, and the rationale behind it, in the methods. It would help the reader if that was included in the Results section.

Response: We revised the Results section to comment on the use of GAL80ts (line 126).

2) " Similar effects were observed using an independent Atro RNAi line (Fig. S1F)." This reads a bit oddly as the paragraph never says which line was used for the other data shown. Maybe just say, as in the previous paragraph, that results were obtained with two different RNAis.

Response: We revised the Results section as suggested (line 134).

3) "When Yki levels were reduced in posterior wing cells by RNAi, the expression of Yki target genes was strongly reduced throughout the posterior compartment (Fig. 1K, Fig. S1G). When knockdown of Atro was combined with knockdown of Yki, the effect of Yki knockdown was partially reversed, as low levels of ex-lacZ and Diap1 were observed throughout the wing pouch (Fig. 1L, Fig. S1H)." This could be clearer, as the partial reversal seems to rest on the difference between "strongly reduced" and "low levels".

Response: We revised the text here to clarify that an intermediate level of expression is observed in the double knockdown (line 165).

4)"was reduced near the center of the wing pouch" Do the authors mean distally? I'm not sure the term center is used previously except in the figure legend. Center can be a bit confusing, as it is a term that means different places in the wing pouch vs the wing blade.
Also, while were on terms for axes, it would help the reader to include a "proximal" tag on the dorsal side of the wing pouch as well as the ventral in Fig. 1B, more consistent with the white arrows used elsewhere in Fig. 1.

Response: We revised the text here to read distally, as suggested (line 170). We also revised Fig 1B as suggested.

5) "As for Atro, this reduction in nuclear Yki was accompanied by increased levels of Ds and reduced junctional levels of Dachs (Fig. S5D,E)." The authors mean I think "As shown previously for Atro". As for often has a different meaning. As for Frank, he loved Trump's changes at the NIH.

Response: We revised the text here to along the lines suggested (line 386).

© 2026. Published by The Company of Biologists under the terms of the Creative Commons Attribution License (https://creativecommons.org/licenses/by/4.0/).

Reviewer 3:

SUMMARY OF THE ADVANCE MADE IN THIS PAPER AND ITS POTENTIAL SIGNIFICANCE TO THE FIELD

In this manuscript Mikalauskaite, et al. investigate the link between Atro and Hippo signaling in Drosophila wing discs. They show - by modulating Atro expression levels - that Atro regulates the expression of Hippo/Yki pathway target genes (diap1 and ex). They also show that modulating Atro levels can rescue the effects (at least partially) of increased or decreased Yki activity in the wing disc. They demonstrate that Atro regulates Hippo pathway signaling by promoting nuclear accumulation of Yki, and that Atro regulates Fj/Vg levels and Dachs localization at junctions. Thus, Atro regulates Hippo signaling by interacting with the Ft-Ds pathway. Lastly, the authors demonstrate that Atro physically binds to Vg, and that this binding likely interferes with Vg-Sd binding. In sum, Mikalauskaite, et al. show that Atro is a multifunctional transcriptional factor that integrates Notch signaling, Vg activity, and Ds-Ft-Hippo signaling to regulate Yki target gene expression. In its current state the manuscript is suitable for publication. The authors did a good job of addressing reviewer concerns/comments and incorporating reviewer recommendations.

Response: Thank-you!

SUGGESTIONS TO AUTHORS

Third decision letter

MS ID#: dev.205540R2

MS Title: Regulation of Hippo signaling by Atrophin in the developing Drosophila wing

Authors: Deimante Mikalauskaite; Cordelia Rauskolb; Tom Lehan; Srividya Venkatramanan; Lilia Carnes; Kenneth D. Irvine

Dear Dr Irvine,

I am happy to tell you that your manuscript has been accepted for publication in Development, pending our standard publication integrity checks.

© 2026. Published by The Company of Biologists under the terms of the Creative Commons Attribution License (https://creativecommons.org/licenses/by/4.0/).
